# Antitumor Effects of Saffron-Derived Carotenoids in Prostate Cancer Cell Models

**DOI:** 10.1155/2014/135048

**Published:** 2014-05-11

**Authors:** Claudio Festuccia, Andrea Mancini, Giovanni Luca Gravina, Luca Scarsella, Silvia Llorens, Gonzalo L. Alonso, Carla Tatone, Ernesto Di Cesare, Emmanuele A. Jannini, Andrea Lenzi, Anna M. D'Alessandro, Manuel Carmona

**Affiliations:** ^1^Laboratory of Radiobiology, Department of Biotechnological and Applied Clinical Sciences, University of L'Aquila, 67100 L'Aquila, Italy; ^2^Section of Medical Pathophysiology, Food Science and Endocrinology, Department of Experimental Medicine, University of Rome “La Sapienza”, 00161 Rome, Italy; ^3^Area of Physiology, Department of Medical Sciences, School of Medicine and Regional Centre for Biomedical Research (CRIB), University of Castilla-La Mancha, 02006 Albacete, Spain; ^4^Cátedra Química Agricola, Universidad de Castilla-La Mancha, Avenida de España s/n., 02071 Albacete, Spain; ^5^Department of Health Sciences, University of L'Aquila, 67100 L'Aquila, Italy; ^6^Department of Biotechnological and Applied Clinical Sciences, Division of Radiotherapy, San Salvatore Hospital, University of L'Aquila, 67100 L'Aquila, Italy; ^7^Department of Clinical and Applied Sciences and Biotechnologies, School of Sexology, University of L'Aquila, 67100 L'Aquila, Italy; ^8^Albacete Science and Technology Park, Avenida de la Innovacion, 02006 Albacete, Spain

## Abstract

*Crocus sativus* L. extracts (saffron) are rich in carotenoids. Preclinical studies have shown that dietary intake of carotenoids has antitumor effects suggesting their potential preventive and/or therapeutic roles. We have recently reported that saffron (SE) and crocin (CR) exhibit anticancer activity by promoting cell cycle arrest in prostate cancer (PCa) cells. It has also been demonstrated that crocetin esters are produced after SE gastrointestinal digestion by CR hydrolysis. The aim of the present report was to investigate if SE, crocetin (CCT), and CR affected *in vivo* tumor growth of two aggressive PCa cell lines (PC3 and 22rv1) which were xenografted in male nude mice treated by oral gavage with SE, CR, and CCT. We demonstrated that the antitumor effects of CCT were higher when compared to CR and SE and treatments reverted the epithelial-mesenchymal transdifferentiation (EMT) as attested by the significant reduction of N-cadherin and beta-catenin expression and the increased expression of E-cadherin. Additionally, SE, CR, and CCT inhibited PCa cell invasion and migration through the downmodulation of metalloproteinase and urokinase expression/activity suggesting that these agents may affect metastatic processes. Our findings suggest that CR and CCT may be dietary phytochemicals with potential antitumor effects in biologically aggressive PCa cells.

## 1. Introduction

The need for anticancer drugs with high efficacy and low toxicity has led to studies evaluating putative antineoplastic factors in fruits, vegetables, herbs, and spices. Natural products are grouped into three main categories: phenylpropanoids, isoprenoids, and alkaloids, which are widely distributed in plant foods and medicinal herbs [1–3] and are crucial to human nutrition and health. Saffron, the dry stigmas of the plant* Crocus sativus *L., belongs to the Iridaceae family and is cultivated in Iran, Spain, Greece, and Italy [4–9]. This is widely known as a spice and its uses in traditional medicine are well established [10, 11]. Major constituents of saffron extracts (SE) belong to the category of carotenoids. The highly water-soluble carotenoids, crocins, are responsible for the majority of its color and represent the major components of SE. The bitter taste of saffron is attributed to picrocrocin, a degradation product of the zeaxanthin carotenoid and also a monoterpene glycoside precursor of safranal. Safranal is an aromatic aldehyde that is the main component of plant volatile oil [12, 13]. Studies in animal models and with cultured human malignant cell lines have demonstrated antitumor and anticancer activities of SE [11, 14–19]. The mechanisms underlying cancer chemopreventive activities of SE include (i) inhibition of synthesis of DNA and RNA, but not protein [16]; (ii) ability to scavenge free radicals [20, 21]; (iii) involvement in the metabolic conversion of carotenoids to retinoids [22]; (iv) direct or indirect interactions with topoisomerase II [16]; (v) promotion of interactions mediated via lectins [23]; and (vi) downregulation of the expression of the catalytic subunit of the human telomerase, hTERT [24].

We have previously demonstrated that SE and CR possessed* in vitro *antiproliferative/cytostatic effects in prostate cancer cells [12] with low proapoptotic activity. Here, two aggressive prostate cancer cell lines, PC3 and 22rv1, were xenografted into nude mice and tumor-bearing animals were treated by oral gavage with saffron extract (300 mg/Kg/day), crocin (200 mg/Kg/day), and crocetin (30 mg/Kg/day) after the development of palpable tumors. The addition of CCT as an additional experimental group was due to the evidence that crocetin esters are produced after SE gastrointestinal CR hydrolysis [24, 25]. Interestingly, we observed that CCT showed the strongest antitumor effects when compared to CR and SE and this was in agreement with the data observed* in vitro*. The major potential mechanism that may explain the antitumor activity of SE and its components CR and CCT is the direct interference with topoisomerase II inducing DNA damage and apoptosis and the reversion of epithelial-mesenchymal transition (EMT), a critical event in the progression of cancer.

## 2.  Material and Methods

### 2.1. Materials

All the materials for tissue culture were purchased from Hyclone (Cramlington, NE, USA). Plasticware was obtained from Nunc (Roskilde, Denmark). Antibodies were purchased from Santa Cruz (Santa Cruz, CA, USA) unless otherwise indicated.

### 2.2. Saffron Spice and Crocin Preparation

Saffron spice harvested from the Cooperativa Altopiano di Navelli company (Navelli, L'Aquila, Italy) during the 2011-2012 harvest was kindly provided by Agenzia per lo Sviluppo, Chamber of Commerce, L'Aquila, Italy [11]. Briefly, 1 g of dried and ground stigma was extracted with 20 mL of water or 85% (v/v) ethanol for 3 h in the dark. The extracts were filtered and concentrated under vacuum, and the extracts were kept at 4°C until use. Crocin was purified by the HPLC method. We used a Varian 9012 liquid chromatographic system equipped with a Varian 9050 UV detector (Walnut Creek, CA, USA). The separations were carried out on a Phenomenex Lichrosphere 5 RP C18 column (250 × 4.6 mm, 5 *μ*m) (Torrance, CA, USA). The precolumn was a Phenomenex C18 column (30 × 4 mm). The detector was set at 442 nm with a spectral acquisition rate of 1.25 scans/s. For the mobile phase, solvent A (methanol) and solvent B (1% [v/v] aqueous acetic acid in water) were used. The mixing of the gradient solvent eluting system was as follows: initial 30% A and 70% B; 0–5 min, linear change to 40% A; 5–10 min, change to 55% A; 10–25 min, change to 68% A; 25–27 min, change to 90% A; 27–30 min, 90% A; 30–33 min, change to 30% A; 33–40 min, 30% A. The flow rate of the mobile phase was 0.8 mL/min, and the injection volume was 20 *μ*L. All solutions were filtered through a 0.2 *μ*m hydrophilic polypropylene membrane (Millipore, Richmond, USA) before use. Separation was accomplished at 25°C. Five different concentrations of crocin solutions were prepared to determine the calibration curve. The calibration curve was constructed with crocin content versus peak area (*y* = 0.0002*x* + 1.0422; *R* ^2^ = 0.9993; linear range: 0.01–0.2 mg/mL). The content of crocin in SE was calculated using the standard curve of crocin, and determinations were repeated three times.

### 2.3. Crocetin Preparation

Saffron spice samples were obtained from the Verdú Cantó Saffron Spain company (Novelda, Alicante, Spain) during the 2011-2012 harvest. The CCT isolation was performed by an internal protected method of the Verdú Cantó Saffron Spain company using the same saffron batch. Quality control of saffron spice was done by UV-vis spectrophotometry according to ISO 3632. Saffron spice aqueous extracts at 500 mg/L concentration were prepared with ultrahigh-purity water. They were magnetically stirred for 1 hour at room temperature in the dark and centrifuged at 4000 rpm for 5 min (Selecta, Barcelona, Spain). Their spectral characteristics were monitored by scanning from 190 to 700 nm using a Perkin-Elmer Lambda 25 spectrophotometer (Norwalk, CT, USA) with UV WinLab 2.85.04 software (Perkin-Elmer) and their coloring strengths (E_1 cm_^1%^ 440 nm, E_1 cm_^1%^ 257 nm, and E_1 cm_^1%^ 330 nm) were determined. Samples of saffron spice and CCT were analyzed by the reverse-phase HPLC technique. Twenty *μ*L of aqueous extracts of saffron spice (500 mg/L) and CCT aqueous solutions (252 mg/L) was filtered through a 0.45 *μ*m PTFE filter and injected into an Agilent 1200 chromatograph (Palo Alto, CA) operating with a 150 mm × 4.6 mm i.d., 5 *μ*m Phenomenex (Le PecqCedex, France) Luna C18 chromatographic column, at 30°C. Eluents were water (A) and acetonitrile (B) with the following elution gradient: 20% B, 0–5 min; 20–80% B, 5–15 min; 80% B, 15–18 min; 20% B, 18–30 min. The flow rate was 0.8 mL min^−1^ and the DAD detector (Hewlett Packard, Waldbronn, Germany) was set at 440 nm for the detection of crocetin esters in saffron spice samples and cis/trans CCT in the samples of CCT isolated from saffron spice [25]. Crocetin esters quantification was estimated using the method based on the extinction coefficient and the related area calculated according to [26, 27].

### 2.4. Cell Cultures

PC3 [28] and 22rv1 [29] cells were obtained from ATCC and DSMZ cell bank, respectively. Cells were seeded at a density of 2 × 10^4^ cells/mL in 24-well plates. Cells were left to attach and grow in 5% FCS DMEM for 24 h. After this time, cells were maintained in the appropriate culture conditions. Morphological controls were performed every day with an inverted phase-contrast photomicroscope (Nikon Diaphot, Tokyo, Japan) before cell trypsinization and counting. Cells were trypsinized and resuspended in 1.0 mL of saline and counted using the NucleoCounter NC-100 (Chemotec, Cydevang, DK) as previously described [30]. NucleoCounter NC-100 also allows determination of the number of dead cells present in a cell sample and, therefore, we considered viable and dead cells to be separated entities. All experiments were conducted in triplicate. IC50 values were calculated by the GraFit method (Erithacus Software Limited, Staines, UK). The effect on cell proliferation was measured by taking the mean cell number with respect to controls over time for the different treatment groups. As a second method for determining the cytotoxicity of PCa cells, we used the crystal violet assay. They were allowed to attach overnight before treatments as described above. After washing with PBS, the cells were incubated and were slightly shaken at RT with 50 *μ*L staining solution (0.5% crystal violet, 20% methanol) that stains DNA. The plate was washed twice with dH_2_O and dried completely. The taken-up crystal violet was solubilized by addition of 200 *μ*L of methanol and 15 min incubation on a shaker. Finally, the amount of dye taken up by the monolayer was quantified by measuring the absorbance at 570 nm in a microplate reader. All studies were performed in triplicate and independently repeated three times.

### 2.5. Mouse Fibroblast NI3T3-Conditioned Medium

Mouse NIH3T3 fibroblast (ATCC) were incubated at 37°C in a humidified atmosphere of 95% air 5% CO_2_ with medium changes every 2 days. For collection of conditioned medium, subconfluent cell cultures were replaced with serum-free medium and left for additional 24 hr at 37°C. Next, medium was collected, centrifuged to eliminated cell debris, and stored at −20°C until use.

### 2.6. Migration and Invasion Assay

Cell migration and invasion assays were performed after appropriate treatments using Boyden chambers containing 8 *μ*m PVPF polycarbonate filters (Nucleopore, Concorezzo, Milan, Italy) coated on one side with 10 *μ*g/mL type I collagen or 25 *μ*g/mL of Matrigel (Becton Dickinson Italia, Milan, Italy), respectively. Tests were performed as previously described [31].

### 2.7. Protease Expression

The expression and activation of gelatinase A (pro-MMP-2) and gelatinase B (pro-MMP-9) were analyzed by zymography performed using SDS-polyacrylamide gel copolymerized with 0.1 mg/mL gelatin [32]. For plasminogen activator analysis, gels were performed by copolymerizing SDS-polyacrylamide with 0.1 mg/mL of lactose-free casein and 15 mg/mL of human plasminogen B as previously described [32]. Routinely, conditioned media were obtained in cultures grown on 24-well plates coated with different substrates. After adhesion, cells were washed with PBS and incubated for different times according to experimental protocols. Following incubation, culture supernatants were collected, centrifuged at top speed in an Eppendorf Microcentrifuge (Hamburg, Germany) for 5 min to remove cell debris, and stored at –80°C until being assayed. Corresponding monolayers were trypsinized and the cells counted in a Neubauer chamber (Hausser, Blue Bell, PA, USA) to normalize the gelatinase activity of the conditioned media.

### 2.8. Western Blotting

Conditioned media from treated and untreated cells were electrophoresed under reducing conditions and transferred to nitrocellulose filter (Schleicher and Schuell GmbH, Dassel, Germany). Nonspecific binding sites were blocked for 1 h in 5% nonfat dried milk in a Tris buffer containing 20 mM Tris and 137 mM NaCl (pH 7.6). Blots were incubated with 1 *μ*g/mL of primary antibody diluted in blocking solution for 1 h at room temperature, before being washed and then incubated for 1 h in secondary antibody diluted in blocking solution according to the manufacturer's protocol. Following a further wash, reactive bands were visualized by chemiluminescent detection (Supersignal, Perbio Science, Tattenhall, UK) using a Bio-Rad gel DocTM (Bio-Rad Laboratories S.r.l., Milan, Italy).

### 2.9. Animals and Therapeutic Regimes

Male CD1 athymic nude mice (Charles River, Calco, Milan, Italy) were maintained under the guidelines established by our institution (University of L'Aquila, Medical School and Science and Technology School Board Regulations, in compliance with the Italian government regulation number 116 on January 27, 1992 for the use of laboratory animals) as previously described [33]. Before any invasive manipulation, mice were anesthetized with a mixture of ketamine (25 mg/mL) and xylazine (5 mg/mL). PC3 or 22rv1 cells (1 × 10^6^ cells/mouse) mixed with Matrigel (Becton Dickinson Labware, Bedford, MA, USA) were injected subcutaneously (s.c.) into the right hind limb of nude mice. At about 10 days after the tumor injection, 48 mice with tumor volume of 0.5–0.8 cm^3^ were retained and randomly divided into four groups (8 mice per group): (1) control; (2) saffron extract (300 mg/kg administered po 5 days/week); (3) crocin (200 mg/kg administered po 5 days/week); (4) crocetin (100 mg/kg administered po 5 days/week).

### 2.10. Evaluation of* In Vivo* Treatment Response

The effects on tumor growth of different treatments were evaluated measuring the following: (1) tumor volume measured during and at the end of the experiment. Tumor volume was assessed by twice a week measurements of tumor diameters with a vernier caliper (length × width). The volume of the tumor was expressed in mm^3^ according to the formula 4/3*πr* ^3^; (2) weight measured at the end of experiment; (3) complete response (CR) defined as the disappearance of a measurable lesion; (4) partial response (PR) defined as a reduction of greater than 50% of tumor volume; (5) stable disease (SD) defined as a reduction of less than 50% or an increase of less than 50% of tumor volume; (6) tumor progression (TP) defined as an increase of greater than 50% of tumor volume; and (7) time to progression (TTP). Tumor growth delay (TGD) was determined as %TGD = ((*T* × *C*)/*C*) × 100, where *T* and *C* are the mean times in days required to reach the same fixed tumor volume in the treated group and control group, respectively [34].

### 2.11. *In Vivo* Toxicity

Serial bodyweight measurements were performed every 3-4 days during treatment. Changes in bodyweight were compared using the control groups and the three treatment groups in each xenograft model.

### 2.12. Manipulation of Tumor Tissue Samples

Animals were euthanized by CO_2_ inhalation and tumors were subsequently removed surgically. Half of the tumor was directly frozen in liquid nitrogen for protein analysis and the other half was fixed in paraformaldehyde overnight for immunohistochemical analyses. Indirect immunoperoxidase staining of tumor xenograft samples was performed on paraffin-embedded tissue sections (4 *μ*m). Briefly, sections were incubated with primary antibodies overnight at 4°C. Next, avidin-biotin assays were done using the Vectastain Elite kit obtained from Vector Laboratories. Mayer's hematoxylin was used as a nuclear counterstain. Negative controls were incubated only with universal negative-control antibodies under identical conditions, processed and mounted. Images of the stained blood vessels were taken using a Leitz photomicroscope. For immunohistochemical analyses we used the following antibodies: monoclonal mouse anti-human E-cadherin, clone NCH-38; monoclonal mouse anti-human N-cadherin, clone 6G11; monoclonal mouse anti-human cytokeratin, clone AE1/AE3; monoclonal mouse anti-human beta-catenin, clone *β*-catenin-1; monoclonal mouse anti-vimentin, clone V9; monoclonal mouse anti-human Ki-67 antigen, clone MIB-1; monoclonal mouse anti-human Ki-67 antigen, clone MIB-1 (Dako Italia, Cernusco sul Naviglio, MI); mouse CD31 (PECAM-1) antibody (Dianova GmbH, Hamburg, Germany); and APO-BrdU TUNEL Assay Kit, (Invitrogen Ltd., Paisley, UK).

### 2.13. Martius Yellow-Brilliant Crystal Scarlet Blue Technique

Stains for these techniques were purchased from HD Supplies (Aylesbury, UK). This technique was used to analyze the presence of red cells dispersed in tumor tissue and present in blood vessels as well as to better evaluate the presence of microthrombi and bleeding zones. Martius yellow, a small-molecule dye together with phosphotungstic acid in alcoholic solution stains red cells. Early fibrin deposits may be colored, but the phosphotungstic acid blocks the staining of muscle, collagen, and connective tissue fibers. Brilliant crystal scarlet, a medium-sized molecule, stains muscle and mature fibrin. Phosphotungstic acid removes any red stain from the collagen. The large-molecule dye aniline blue stains the collagen and old fibrin.

### 2.14. Statistics

Continuous variables were summarized as mean and standard deviation or as median and 95% CI for the median. For continuous variables, statistical comparisons between control and treated groups were established by carrying out the Kruskal-Wallis test. When the Kruskal-Wallis test revealed a statistical difference, pairwise comparisons were made by the Dwass-Steel-Chritchlow-Fligner method and the probability of each presumed “nondifference” was indicated. Dichotomous variables are summarized by absolute and/or relative frequencies. For dichotomous variables, statistical comparisons between control and treated groups were established by carrying out the exact Fisher's test. For multiple comparisons, the level of significance was corrected by multiplying the *P* value by the number of comparisons performed (*n*) according to the Bonferroni correction. TTP was analyzed by Kaplan-Meier curves and Gehan's generalized Wilcoxon test. When more than two survival curves were compared, the log-rank test for trend was used. This tests the probability that there is a trend in survival scores across the groups. All tests were two-sided and were determined by Monte Carlo significance. *P* values <0.05 were considered statistically significant. SPSS (statistical analysis software package) version 10.0 and StatDirect (version. 2.3.3, StatDirect Ltd.) were used for statistical analysis and graphic presentation.

## 3. Results

### 3.1. Effect of SE, CCT, and CR on Tumor Growth in Athymic Nude Mice

**Figure 1:**
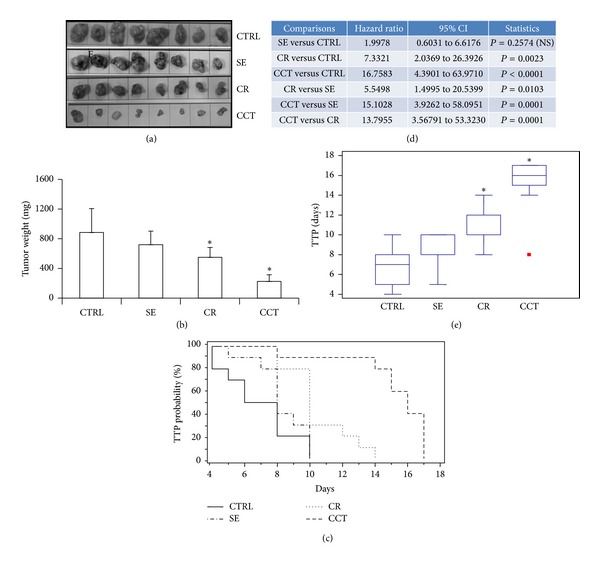
*In vivo *experiments using PC3-bearing male nude mice treated with saffron extract (SE, 300 mg/kg administered po 5 days/week), crocin (CR, 200 mg/kg administered po 5 days/week), and crocetin (CCT, 100 mg/kg administered po 5 days/week) administered by oral gavage when tumors reached 0.5–0.8 cm^3^. After 30 days of treatment, animals were sacrificed and tumors harvested, weighed, and analyzed. Experiments were performed using 8 animals/group and were stopped at the indicated times. (a) Macroscopic appearance of PC3 tumors subjected to different treatments. (b) Tumor weight comparisons at the end of experiments. CR and CCT induced a significant reduction of tumor weight in the PC3 xenografts whereas SE resulted in only a 20% reduction. (c) Time to progression (TTP) probability determined by Kaplan-Meier analysis in PC3 xenografts. (d) Hazard ratio value with 95% CI and *P* values determined according to different treatments. (e) Comparisons of TTP expressed in days after treatments.

**Figure 2:**
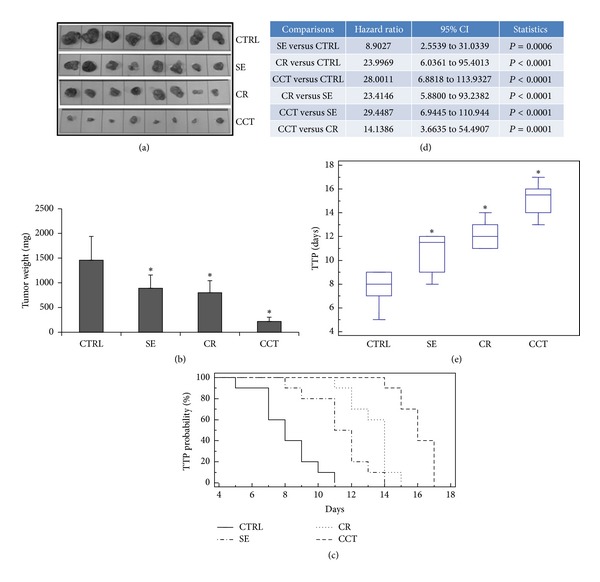
*In vivo *experiments using PC3-bearing male nude mice treated with saffron extract (SE, 300 mg/kg administered po 5 days/week), crocin (CR, 200 mg/kg administered po 5 days/week), and crocetin (CCT, 100 mg/kg administered po 5 days/week) administered by oral gavage when tumors reached 0.5–0.8 cm^3^. After 30 days of treatment, animals were sacrificed and tumors harvested, weighed, and analyzed. Experiments were performed using 8 animals/group and were stopped at the indicated times. (a) Macroscopic appearance of PC3 tumors subjected to different treatments. (b) Tumor weight comparisons at the end of experiments. CR and CCT induced a significant reduction of tumor weight in the PC3 xenografts whereas SE resulted in only a 20% reduction. (c) Time to progression (TTP) probability determined by Kaplan-Meier analysis in PC3 xenografts. (d) Hazard ratio value with 95% CI and *P* values determined according to different treatments. (e) Comparisons of TTP expressed in days after treatments.

Tumors were measured twice per week. We observed that SE, CR, and CCT reduced tumor growth in both PC3 and 22rv1 (Figures 1 and 2) xenografts confirming our previous* in vitro* data [12]. In the PC3* in vivo* model, the tumor mass (Figures 1(a)–1(c) and Table 1) was, respectively, reduced by 18% (*P* > 0.05), 38% (*P* < 0.05), and 75% (*P* < 0.001) after SE, CR, and CCT treatment with respect to controls. The comparative effect of treatment with extract (SE) versus crocin (CR) in terms of tumor growth did not reach statistical significance (*P* > 0.05) whereas crocetin (CCT) significantly affected tumor growth with respect to SE and CR (*P* < 0.001). TTP probability, as determined by Kaplan-Meier analyses, showed a delay of tumor progression with respect to control in all treatments except for SE (Figures 1(c)–1(e)). The analysis of hazard ratio (HR) values indicated that CR- and CCT-based treatments significantly improved the probability of delaying the tumor progression (Figure 1(d)), with the best performance observed for CCT. This evidence paralleled with data of panel E in which CR- and CCT-based treatments significantly increased the median time necessary to tumor progression. SE treatment significantly delayed the occurrence of tumor progression although to a lesser extent with respect to CR and CCT.

In the 22rv1* in vivo* model, the tumor mass (Figures 2(a)–2(c) and Table 2) was, respectively, reduced by 33% (*P* < 0.05), 35% (*P* < 0.05), and 80% (*P* < 0.001) after SE, CR, and CCT treatment with respect to controls (Figure 2(b)). Therefore, when the two xenograft models were compared in terms of tumor growth reduction, all treatments were more effective in the 22rv1 tumor model. TTP probability, as determined by Kaplan-Meier analyses, showed a delay of tumor progression with respect to control in all treatments except for SE (Figures 2(c)–2(e)). The analysis of hazard ratio (HR) values indicated that CR- and CCT-based treatments significantly improved the probability of delaying the tumor progression (Figure 2(d)), with the best performance observed for CCT. This evidence paralleled with data of panel E in which CR- and CCT-based treatments significantly increased the median time necessary to tumor progression (Figure 2(e)). SE treatment significantly delayed the occurrence of tumor progression although to a lesser extent with respect to CR and CCT (Figures 2(c)–2(e)).

Upon SE, CR, and CCT treatment (Table 1), the proliferation index (PI) of the PC3 tumor model, assessed by Ki67, was lower by 16% (*P* > 0.05), 30% (*P* < 0.01), and 59% (*P* < 0.001) with respect to controls. SE, CR, and CCT treatment (Table 2) decreased the percentage of Ki67 by 15% (*P* > 0.05), 27% (*P* < 0.05), and 60% (*P* < 0.001) with respect to control.

SE, CR, and CCT treatments (Table 1) decreased the vessel count (CD31-positive endothelial cells) by 2% (*P* > 0.05), 12% (*P* > 0.05), and 30% (*P* < 0.01) with respect to control, while in the 22rv1 tumor model the vessel count was reduced by 18% (*P* > 0.05), 29% (*P* < 0.05), and 44% (*P* < 0.001) (Table 2). Apoptosis, as assessed by TUNEL assay, significantly increased after CCT treatment (*P* < 0.001) in both tumor models while the SE and CR treatments did not significantly affect apoptosis (Tables 1 and 2).

**Figure 3:**
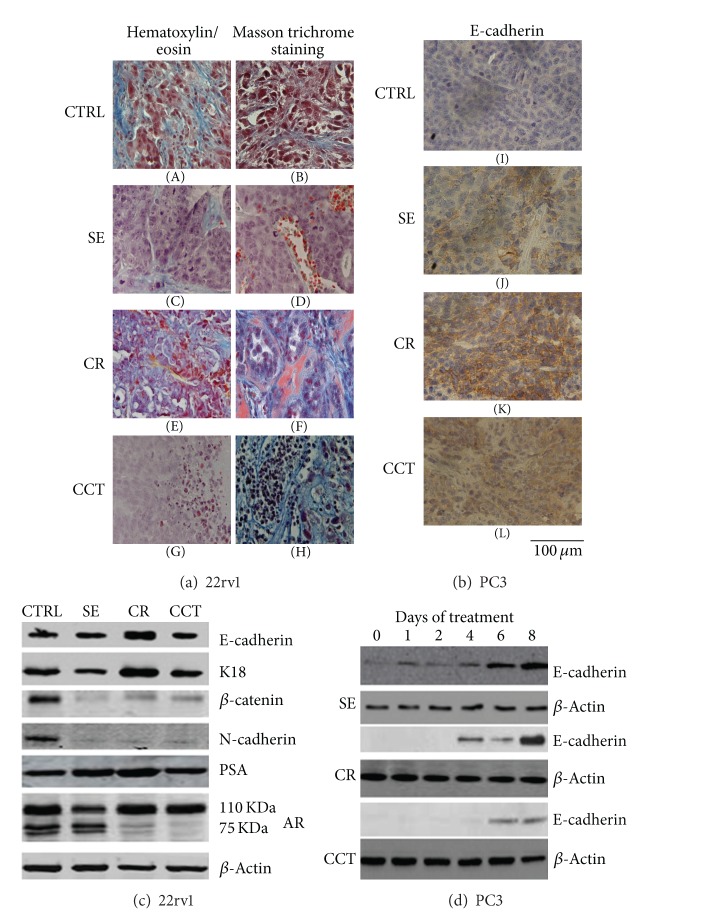
(a) Hematoxylin/eosin (A, C, E, G) and Masson trichrome (B, D, F, H) staining of 22rv1 tumors treated with SE (C, D), CR (E, F), and CCT (G, H). Untreated 22rv1 tumors show tumor cell nests enveloped by dense blue-stained collagen deposits that are abundant in the peripheral areas (A). Tumor cell nests show their undifferentiated characteristics (B). SE seem to induce a differentiated phenotype with large tumor cells and prominent nuclei organizing in structures similar to glands (C, D). Vessels (D) appear dilated with unstructured capillary bed dispersed in collagen I deposits (azure staining). The differentiated appearance was greater after CR treatment (E, F) with smaller blood pervious vessels that follow the fibrous strands of collagen and surrounding the pseudoglandular acini (E). CR treatment induces deposition of fibrin clots (pale pink/orange staining) dispersed in dense collagen I deposits (fibrosis), enveloping tumor cell nests, as evolution of massive blood pouring. This appearance was not associated with presence of thrombotic vessels. CCT treatments (G, H) show a histological structure poorly differentiated (G) and rich in necrotic areas with the presence of numerous phagocytes (neutrophils and monocytes) dispensed in collagen I deposit resulting from a colliquative necrosis. Scale bar is 100 *μ*m. (b) Immunohistochemical evaluation of E-cadherin in PC3 xenografts (I, J, K, L) treated or not with SE (J), CR (K), or CCT (L). Indirect immunoperoxidase staining of tumor xenograft samples was performed on paraffin-embedded tissue sections (4 *μ*m). It is opportune to note the strong E-cadherin staining after SE and CR treatments whereas controls (CTRL) tissues were negative and CCT showed low levels of this antigen. Scale bar is 100 *μ*m. (c) Western blotting performed on 22rv1 cell extracts derived from cultures treated for 8 days with SE (0.4 mg/mL), CR (0.4 mM), and CCT (0.1 mM). EMT markers (vimentin, *β*-catenin, and N-cadherin) were significantly reduced whereas epithelial markers (E-cadherin, K18, and PSA) were upregulated in 22rv1 cells. Interestingly, the expression levels of the truncated form (ligand-independent) of AR were significantly reduced after CR and CCT treatments whereas no changes were showed for full-length AR levels. (d) Densitometric analysis performed on E-cadherin and K18 expression in PC3 and 22rv1 cells treated with SE, CR, and CCT. (d) Time-dependent modulation of E-cadherin after treatments with SE (0.4 mg/mL), CR (0.4 mM), and CCT (0.1 mM) in the PC3 cell model (an* in vitro* assay). Western blots are representative of three different analyses.

Histochemical staining with hematoxylin/eosin, Masson trichrome, and Martius yellow-brillant crystal scarlet blue were used to analyze potential morphological changes treatments induced by treatments. Dense blue-stained collagen I deposits, suggestive of fibrosis, enveloped tumor cell nests in peripheral areas whereas PCa cells were arranged to form large cell masses surrounded by red-stained dilated blood vessels. Blue-stained collagen strands followed tumor cell growth but entrapped tumor nests in treated cells often in areas in which necrosis was also present. These behaviors were more evident in tissue sections derived from xenografts harvested from mice treated with CR and CCT. In these mice, xenograft tumors had massive fibrosis and orange-stained fibrin strands, which appeared right where there were residues of blood vessels or blood pouring. In Figure 3(a), we show representative microscopic fields demonstrating histological effects of SE, CR, and CCT treatments in 22rv1 tumors.

### 3.2. Differentiation Effects of SE and Crocin

An important aspect of treatment with carotenoids is the potential for prodifferentiating effects. Literature data suggest that epithelial tumor cell aggressiveness is characterized by epithelial-mesenchymal transdifferentiation (EMT) with aggressive cancers characterized by loss of cell-cell adhesion, repression of E-cadherin and cytokeratin 18 (K18), and increased cell mobility. The major markers for EMT are N-cadherin and *β*-catenin. We analyzed the expression of these antigens by immunohistochemistry in tissues slides harvested from the mice bearing tumor xenografts and treated or untreated with SE, CR, and CCT. These* in vivo* data were coupled with* in vitro* experiments performed on the same tumor cells in the presence of the same treatments. We observed that SE and CR strongly induced E-cadherin and K18 expression with reduced expression of vimentin, N-cadherin, and *β*-catenin. In Figure 3(b), we show a representative staining of E-cadherin in PC3 xenografts upon SE, CR, or CCT treatment. K18 staining had a similar trend whereas vimentin, N-cadherin, and *β*-catenin expression was inverted with high levels in untreated tumors and low or absent expression in treated tumors (data not shown). To directly test the prodifferentiating effects of SE, CR, and CCT, an* in vitro *assessment of EMT markers was performed by using both 22rv1 and PC3 tumor models. Cells were treated with SE (0.4 mg/mL), CR (0.4 mM), and CCT (0.1 mM). After prolonged treatment with these pharmacological agents, a differentiated phenotype was observed in both cellular models. In Figure 3(c), we demonstrate that E-cadherin and K18, two well-known epithelial markers, were significantly increased after CR treatment whereas changes in the extent of these markers were lower after SE. In Figure 3(d), we show the results of densitometric analysis performed on E-cadherin and K18 expression in PC3 and 22rv1 cells treated with SE, CR and CCT. These effects were lower in PC3 cells when compared to those observed in 22rv1 cells. Western blots performed on PC3 cell extracts demonstrate that treatments induced E-cadherin expression in a time-dependent manner. Our results suggests that the induction of a differentiated phenotype may concur in inducing the antitumor properties especially after CCT treatment. The EMT was also investigated by analyzing the expression of vimentin, K18, N-cadherin, *β*-catenin, prostate specific antigen (PSA), and androgen receptor (AR) after 8 days of treatment with SE, CT, and CCT treatments. Mesenchymal markers (*β*-catenin and N-cadherin) changes were of opposite tendency with marked reduced expression upon all treatments. The analyses of markers historically considered to be associated with more differentiated PCa (PSA and AR) showed that SE and CR upmodulated PSA whereas the expression levels of the ligand-independent truncated AR form were significantly reduced after CR and CCT treatment. No changes in the full length AR form were found after SE, CR, and CCT treatments.

### 3.3. Saffron Extract and Its Components Inhibit Cell Invasion through Modulation of Metalloproteinases and Urokinase Expression/Activity

**Figure 4:**
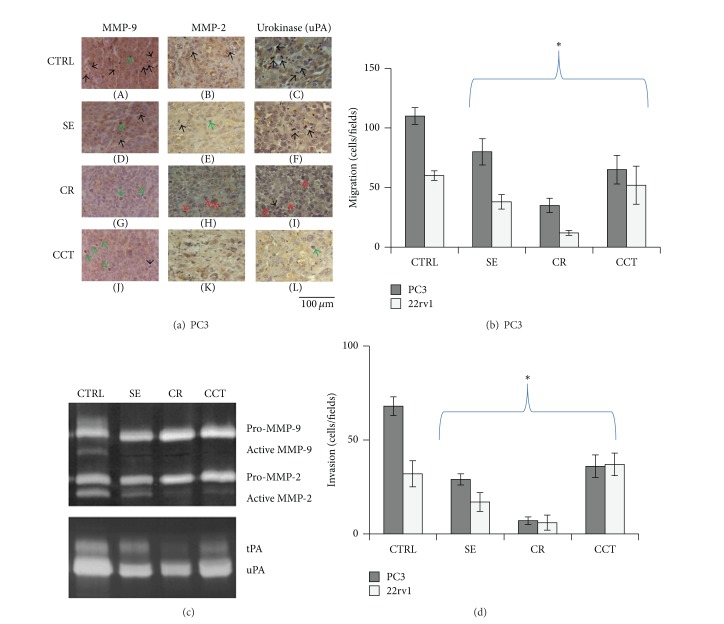
(a) Immunostaining for MMP-9 (A, D, G, J), MMP-2 (B, E, H, K), and uPA (C, F, I, L) performed in PC3 tumors treated or not with SE (D, E, F), CR (G, H, I), and CCT (J, K, L). Untreated PC3 tumors (A, B, C) show high expression of MMP-9, MMP2, and uPA. Indirect immunoperoxidase staining of tumor xenograft samples was performed on paraffin-embedded tissue sections (4 *μ*m). SE, CR, and CCT significantly reduced the expression of these proteases. Black arrows show mitotic figures (mean 16 ± 4 mitotic figures/100x microscopic field), which are high in CTR and were reduced after SE (about 40% versus CTRL), CR (70% versus CTRL) or CCT (90% versus CTRL). Green arrows show condensed/pyknotic nuclei. This appearance is evident in SE- (1–4 pyknotic nuclei/100x microscopic field), CR- (2–8 pyknotic nuclei/100x microscopic field), or CCT-treated tumor bearing mice (>10 pyknotic nuclei/100x microscopic field). Red arrows show aberrant mitosis, which is more evident in CCT-treated tumors. Scale bar is 100 *μ*m. (b) Zymography for gelatinases, (a) and (b), and plasminogen activator performed in PC3 cells treated with SE (0.4 mg/mL), CR (0.4 mM), and CCT (0.1 mM). Cell cultures were allowed to grow in complete medium for 8 days in presence of SE, CR, and CCT. Next, medium was changed and serum-free medium containing SE, CR, and CCT was added for additional 24–48 hr. (c) Migration assay performed by using filters with 8 *μ*m pores coated with 0.1% gelatin and using as chemoattractant the NI3T3-conditioned medium. Cells were cultured for 8 days in complete medium with SE, CR, and CCT, then harvested by trypsinization, and added to the upper compartment of the Boyden chambers at the above-mentioned concentrations. Five 10x microscopic fields were counted for each replicate. Data (±SD) are representative of three different analyses. Treatment reduced significantly the migration of PC3 and 22rv1 cells (*P < 0.01). (d) Invasion assay performed by using filters with 8 *μ*m pores coated with 50 *μ*L (12.5 *μ*g/mL) Matrigel and using as a chemoattractant the mouse fibroblast NI3T3-conditioned medium. Cells cultured for 6 days with SE, CR, and CCT were added to the upper compartment of Boyden chambers at the above-mentioned concentrations. Five 10x microscopic fields were counted for each replicate. Data (±SD) are representative of three different analyses. We considered *P* < 0.05 as significant (*).

An important aspect of EMT is acquisition of higher migration and invasion capacities of tumor cells coupled with increased expression of proteolytic enzymes of the extracellular matrix (ECM) such as gelatinases A and B, MMP-9, MMP-2, and urokinase-type plasminogen activator (uPA). Therefore, in order to reinforce the concept that saffron-derived compounds maintain a differentiated state of prostate cancer cells, we analyzed the immunohistochemistry expression of proteases involved in the migratory and invasive properties of PCa. PC3 and 22rv1 xenografts were studied after SE (0.4 mg/mL), CR (0.4 mM), and CCT (0.1 mM) treatment. In parallel, we verified the* in vitro* secretion of these metalloproteinases upon the same treatments. Basally, PC3 tumor xenografts are strongly positive for MMP-9, MMP-2, and uPA. Immunohistochemical analyses revealed that MMP-9, MMP-2, and uPA expression levels were markedly reduced after all treatments in both* in vivo* tumor models. In Figure 4(a), we show a representative immunohistochemistry performed on a PC3 xenograft. As for the experiments performed to detect EMT/epithelial marker changes,* in vitro* experiments on PC3 cells were performed by treating these cells for 8 days with SE (0.4 mg/mL), CR (0.4 mM), and CCT (0.1 mM). In order to collect conditioned medium on which we tested metalloproteinase and urokinase activities, cell cultures were allowed to grow in complete medium for 6 days in presence of SE, CR, and CCT. Next, medium was changed and serum-free medium containing SE, CR, and CCT was added for additional 24–48 hr. In Figure 4(b), we show that the active isoforms of MMP-9, MMP-2, the 80 kDa tissue-derived plasminogen activator (tPA), and uPA (47–52 kDa plasminogen activator isoforms) were markedly reduced after treatments. For migration/invasion tests (panels C, D) cells were cultured for 8 days in complete medium, then harvested by trypsinization, and used for the tests.

Interestingly, SE and CR significantly reduced the migratory and invasive properties of PC3 and 22rv1 tumor cells (Figures 4(c)-4(d)). No significant changes were found in either model when tumor cells were treated with CCT (Figures 4(c)-4(d)). These findings fit with the data concerning the protease expression suggesting that SE and CR significantly decrease some key aspects of the metastatic processes.

## 4. Discussion

Prostate cancer continues to be a leading cause of cancer death in European males [35]. Patients with localized prostate cancer possess a favorable 5-year survival rate, whereas patients with metastatic cancer have a median survival of only 12–15 months, which is an indication that PCa cell metastasis is the primary mediator of mortality for this disease [36]. A variety of therapeutic strategies are utilized for the treatment of advanced metastatic PCa [37]. However, despite advances in the understanding of PCa biology, these therapies rarely produce significant increases in survival time in metastatic PCa. Natural products have long been used to prevent and treat diseases including cancers and might be good candidates for the development of anticancer drugs. Many herbs and spices are the subject of scientific investigations related to antioxidant properties and health. Saffron, a commonly used spice and food additive, is known for its anticancer and antitumor properties [12–16, 38]. CR and CCT, two carotenoid compounds derived from saffron, have shown a significant inhibitory effect on the growth of cancer cells [4, 7, 11, 14, 38, 39].

Many mechanisms of action have been identified or supposed. Different hypotheses for antitumor effects of saffron and its ingredients have been proposed including (a) inhibition of DNA and RNA synthesis, but not protein [40]; (b) ability to scavenge free radicals [24]; (c) involvement in the metabolic conversion of carotenoids to retinoids [41]; (d) mediation of interactions of carotenoids with topoisomerase II (an enzyme involved in cellular DNA-protein interaction) [41]; and (e) downregulation of the expression of the catalytic subunit of the human telomerase (hTERT) [42].

Here, we demonstrate that, although different phytochemicals in saffron extracts could have additive and synergistic effects enhancing its anticarcinogenic properties [39], only CR and CCT showed higher antitumor effects with respect to SE. The* in vivo* effects of these compounds were evident in* in vivo* models. SE, CR, and CCT inhibited tumor cell proliferation of aggressive models of PCa as shown by the reduced proliferating cell nuclear antigens, mitotic figure counts, tumor vessels, and tumor growth rate and increased apoptosis especially in the CCT-treated animal group. CCT showed the strongest antitumor effects since it resulted in a tumor weight reduction of 75% and 85% in PC3 and 22rv1 xenografts, with respect to control while CR and SE resulted in tumor weight reduction of 38% and 18%, and 54% and 39%, in PC3 and 22rv1 xenografts Additionally, CCT tumor xenografts had a reduction of 50% and 69% of proliferating index (PI) in PC3 and 22rv1 xenografts, while CR and SE decreased PI by 30% and 16% and 27 and 15%, respectively The induction of apoptosis and the decrease of the vessel count were once again mainly affected by CCT. The changes in biologically relevant markers were parallel with the increase in the time to progression as shown by the data concerning the time trend of tumor growth. Conversely, SE and CR induced stronger epithelial differentiation with increased E-cadherin and K18 expression levels and a decrease in EMT markers such as N-cadherin, *β*-catenin, and vimentin.

EMT phenotype is associated with the activation of the Wnt signaling pathway, in which its key component *β*-catenin plays critical roles in embryonic development as well as in human diseases, including cancer. The progression of carcinomas is associated with the loss of epithelial morphology and a concomitant acquisition of a more mesenchymal phenotype, which in turn is thought to contribute to the invasive and/or metastatic behavior of the malignant process. Loss of E-cadherin is reported to be associated with a poor prognosis [1–6]. It has been demonstrated that N-cadherin was not expressed in normal prostate tissue; however, in prostatic cancer, N-cadherin was expressed in the poorly differentiated areas, which showed negative E-cadherin staining. Accumulated evidence has demonstrated a significant role for the Wnt pathway in the development and progression of human prostate cancer. Clearly, the mere loss of cell-cell contact and communication cannot be the sole explanation for the observed relationship between loss of E-cadherin-mediated adhesion and poor clinical outcome. Recently, a number of papers have been published that describe the inappropriate expression of nonepithelial cadherins by epithelial cells as a putative novel mechanism for promoting the interaction with the stroma, thereby facilitating invasion and metastasis [13–16]. We showed that human prostate cancer cell lines, which lack expression of either E-cadherin or catenins and, therefore, lack an E-cadherin-mediated cell-cell adhesion, acquire cadherin expression [17].

In our previous report we noticed that SE and CR were effective* in vitro* in PCa tumor cells including PC3 cells used in the present report [12]. Androgen sensitive PCa cells seem to be more affected by treatments of androgen insensitive ones. The lower efficacy of SE observed* in vivo*, especially for PC3 cells, could be due to the reduced blood levels (and thus reduced tumor levels) of carotenoids that were achieved after the oral administration of SE. It has been demonstrated that CR was absorbed in the intestinal tract in a minimal amount whereas, when we administered SE* in vitro*, we provide to cultures mainly CR and not CCT which is the metabolic product of CR in gastrointestinal environment. CCT could be, indeed, the major compound which can be found in the blood. Crocin shows intrinsic antiproliferative effects but the oral administration shows only the effects of CCT. In addition, not all crocin turns into crocetin. However the relative blood amounts and blood peaks are unknown. Further experiments are necessary to establish the amount of carotenoids found in the blood after oral administration of SE, CR, and CCT (paper in preparation). In addition, although CCT is more effective of CR, its tumor levels were probably not sufficient to determine antiproliferative/proapoptotic effects in our PCa xenografts.

In addition, as widely described in this paper, CCT showed higher effects on proliferation/apoptosis when compared to differentiative ones; therefore, although MMPs was reduced (whereas uPA was not modified) overall Matrigel degradation (and invasion) seems to be not influenced.

To our knowledge, this is the first report showing that saffron-induced antitumor effects may affect EMT processes. Specifically, we observed that the induction of epithelial differentiation was a time-dependent event and was evident from 4 days of treatment with SE and CR. Based on the current data, saffron and its ingredients could be considered as a promising candidate for clinical anticancer trials in aggressive prostate cancer with a high risk of metastases.

## Figures and Tables

**Table 1:** Antitumor activity of SE, CR, and CCT in PC3 xenografts.

Drug	Dose mg/Kg	Tumors	Weight of mice mean ± SD	Tumor weight (mg ± SD)	PI (ki67 %) mean ± SD	Apoptosis mean ± SD	Vessels mean ± SD
Saline		8	24.0 ± 2.1	885 ± 321	37.3 ± 4.5	<2	30.0 ± 5.5
SE	300	8	26.1 ± 2.0	720 ± 183	31.4 ± 2.7	<2	29.4 ± 2.0
% versus baseline				18.0%	16%		2%
CR	200	8	23.0 ± 1.7	550 ± 132	26.0 ± 3.0	<2	26.4 ± 1.5
% versus baseline				38.0%	30%		12%
CCT	100	8	19.3 ± 1.5	225 ± 91	15.2 ± 1.2	24.0 ± 3.0	18.3 ± 2.5
% versus baseline				75.0%	59%		30%

Apoptosis was measured as the percentage of tunel positive cells ± SD mesured on five random fields (100X); tumor microvessels were counted in five arbitrary selected fields/tumor and the data are presented as number of CD31+ microvessels/microscopic field for each group (100X). Proliferation index (PI) was determined as percentage by counting on 500 cells at 100X the number of Ki67 stained cells.

**Table 2:** Antitumor activity of SE, CR, and CCT in 22rv1 xenografts.

Drug	Dose mg/Kg	Tumors	Weight of mice mean ± SD	Tumor weight (mg ± SD)	PI (ki67 %) mean ± SD	Apoptosis mean ± SD	Vessels mean ± SD
Saline		8	24.3 ± 1.8	1458 ± 481	45.5 ± 6.5	<2	32.7 ± 4.3
SE	300	8	26.1 ± 2.0	890 ± 267	38.7 ± 2.7	<2	26.2 ± 2.7
% versus baseline				39.0%	15%		18%
CR	200	8	23.0 ± 1.7	800 ± 244	32.2 ± 3.3	5.2 ± 2.7	23.2 ± 4.5
% versus baseline				45.1%	27%		29%
CCT	100	8	19.3 ± 1.5	217 ± 85	18.2 ± 1.4	21.5 ± 3.2	18.3 ± 2.5
% versus baseline				85.1%	60%		44%

Apoptosis was measured as the percentage of tunel positive cells ± SD mesured on five random fields (100X); tumor microvessels were counted in five arbitrary selected fields/tumor and the data are presented as number of CD31+ microvessels/microscopic field for each group (100X). Proliferation index (PI) was determined as percentage by counting on 500 cells at 100X the number of Ki67 stained cells.
